# Utility of liquid-based cytology and cell block obtained by vitrectomy under infusion to diagnose vitreoretinal lymphoma

**DOI:** 10.1007/s10384-026-01389-2

**Published:** 2026-06-30

**Authors:** Rintaro Ohe, Sonoka Oyanagi, Eriko Inamura, Takumi Kitaoka, Takanobu Kabasawa, Kazushi Suzuki, Naoya Uchiyama, Ami Shida, Toshinori Suzuki, Keita Togashi, Yutaka Kaneko

**Affiliations:** 1 Department of Pathology Faculty of Medicine, Yamagata University, Yamagata, Japan; 2 Department of Ophthalmology and Visual Sciences Faculty of Medicine, Yamagata University, 2-2-2 Iida-Nishi, Yamagata, 990-9585 Japan; 3 Department of Pathology, Yonezawa City Hospital, Yonezawa, Japan; 4 Department of Pathology, Yamagata University Hospital, Yamagata, Japan

**Keywords:** Vitreoretinal lymphoma (VRL), Vitrectomy under infusion, Liquid-based cytology, Cell block

## Abstract

**Purpose:**

To confirm the definitive diagnostic method of vitreoretinal lymphoma by vitrectomy under infusion.

**Study design:**

Retrospective, pathological analysis

**Methods:**

Twenty-two patients participated in this study; 29 vitreous samples were obtained under infusion. Liquid-based cytology (LBC) was performed by targeting areas with a high cell concentration while observing the surgical field under infusion. Cell block analysis was performed by the vitreous cell components in the intraocular irrigating fluid.

**Results:**

Our vitreous sample collection did not result in adverse events. In LBC samples, atypical large lymphoid cells were identified in 89.7%. These samples contained between 30 and over 10,000 atypical large lymphoid cells. Necrosis was observed in 76.9% in the background of LBC samples. In cell block samples, atypical large lymphoid cells were identified in 87.0%. Immunohistochemical staining revealed the following immunophenotypes for the lymphoid cells: CD20 (100%), CD79a (100%), CD10 (0%), BCL6 (57.1%), MUM1 (85.7%), and Ki-67 LI 66.3±12.7%.

**Conclusion:**

This method ensures safety and high diagnostic accuracy, thereby contributing to the definitive diagnosis of vitreoretinal lymphoma.

**Supplementary Information:**

The online version contains supplementary material available at https://doi.org/10.1007/s10384-026-01389-2.

## Introduction

Primary intraocular lymphoma (PIOL) is considered to include both primary vitreoretinal lymphoma (VRL) and primary uveal lymphoma. The disease-specific survival rates of PIOL are reported as 74.2% at 5 years and 61.5% at 10 years [1]. VRL is known to have a poor prognosis because almost all primary VRLs are diffuse large B-cell lymphoma, an aggressive lymphoma [2, 3]; on the other hand, uveal lymphoma is considered to follow an indolent course because it corresponds to extranodal marginal zone B-cell lymphoma [4]. Furthermore, central nervous system lymphoma may subsequently develop even after a diagnosis of primary VRL [5]. Thus, accurate diagnosis while VRL remains confined to the eye is essential.

The diagnosis of VRL is difficult because obtaining the necessary vitreous sample is technically challenging. The conventional method for obtaining pure vitreous samples involves performing a vitreous biopsy with scleral indentation [6], without irrigation. This method makes intraocular pressure control difficult and carries a risk of vision disorders in the affected eye. Furthermore, this method allows for the collection of only a tiny vitreous sample.

This study describes a VRL diagnostic method using vitreous samples collected by vitrectomy under infusion, unlike the conventional method with scleral indentation [6] without irrigation. This method reduces the risk of complications to the affected eye. Furthermore, we obtained liquid-based cytology (LBC) samples under direct visualization of the surgical field and prepared cell blocks from perfusion fluid that would otherwise be discarded. LBC is known to provide high-quality cell fixation and to emphasize cell-nuclear details [7], thereby providing higher sensitivity and specificity compared to conventional smears [8, 9]. Immunohistochemistry of cell block specimens is reported to be useful for VRL diagnosis [10, 11]. This study demonstrates that the morphological and immunohistochemical findings obtained from the LBC samples and cell blocks would facilitate the establishment of a definitive diagnosis of VRL.

## Methods

### Patients and eyes

This study examined 969 uveitis patients treated at Yamagata University Hospital between 2015 and 2025, identifying vitreous opacity in 86 cases. Among them, patients who showed diffuse vitreous opacity in fundus examinations and demonstrated resistance to anti-inflammatory treatment including steroids, or patients already diagnosed with malignant lymphoma involving multiple organs, were defined as suspected VRL patients. Patients with snowball opacity on fundus examination and periphlebitis on fluorescein angiography, or those already diagnosed with sarcoidosis, were defined as suspected ocular sarcoidosis patients. Patients with sympathetic ophthalmia, viral infections, or fungal infections were defined as other patients. Consequently, among the 86 cases with vitreous opacity, 22 were classified as suspected VRL cases, 23 as suspected ocular sarcoidosis cases, and 41 as other cases. Furthermore, LBC and cell block examinations were performed on 29 eyes from 22 suspected VRL cases, 32 eyes from 23 suspected ocular sarcoidosis cases, and 45 eyes from 41 other cases. Clinically, VRL and ocular sarcoidosis required differential diagnosis, so that the diagnostic method for ocular sarcoidosis in our method has already been reported [12]. This study focuses on the detailed examination of the 29 eyes in the 22 patients suspected of VRL.

**Fig. 1:**
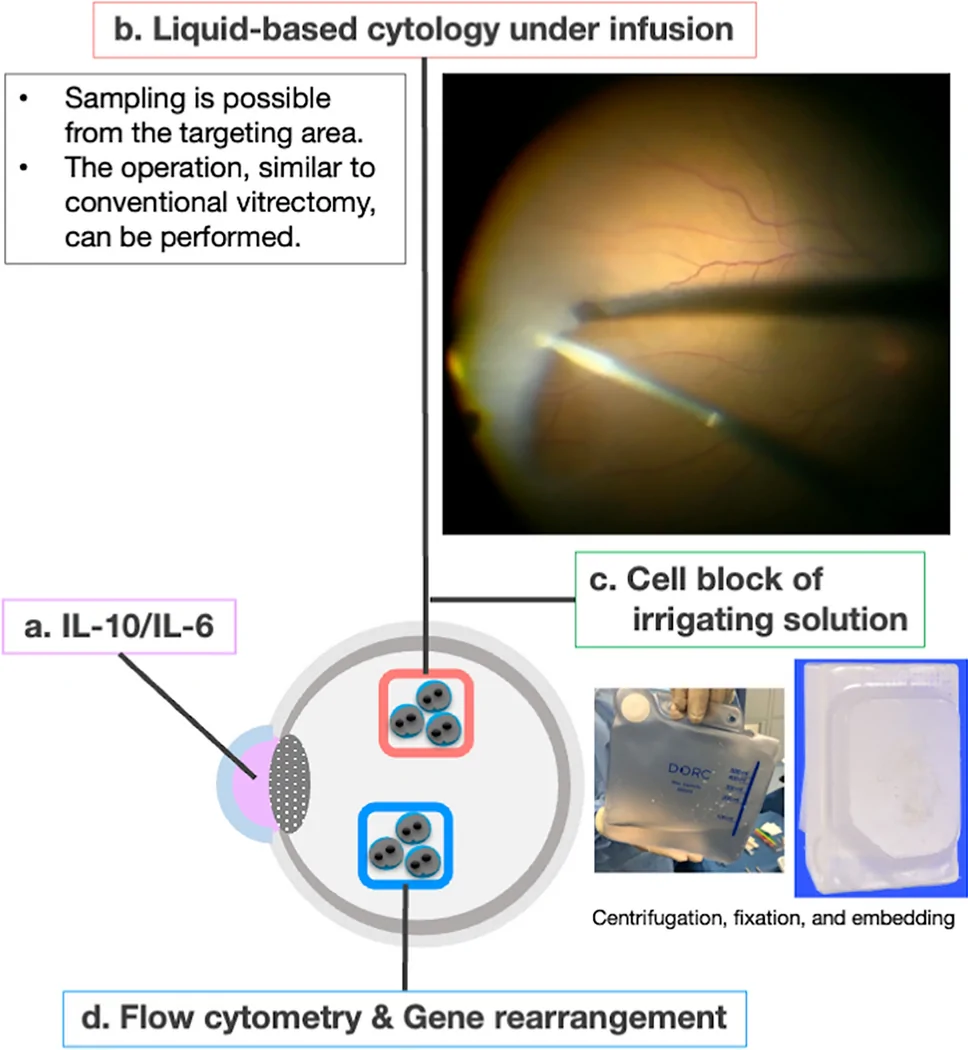
Sample collection method from the vitreous body in this study a. IL-10/IL-6 is measured by pure anterior chamber fluid collected before vitrectomy. b. The samples required for liquid-based cytology are prepared by targeting areas with a high concentration of cells while observing the surgical field under infusion. **c.** The samples required for cell block are prepared by the vitreous cell components in the intraocular irrigating fluid. **d.** The samples required for flow cytometry and gene rearrangement are prepared after cytology, targeting areas with high cell concentrations while observing the surgical field under infusion.

Twenty-nine eyes of 22 patients, presumed to be VRL, were clinically diagnosed by diffuse vitreous opacity in fundus examination (Fig. 1). The specimen collection procedure involved first collecting aqueous humor for cytokine measurement (Fig. 1a). Vitreous samples were collected by vitrectomy under infusion for LBC and cell block preparation (Fig. 1b, c). Additionally, vitreous samples were collected for flow cytometry and gene rearrangement analysis (Fig. 1d). Detailed methods are described below. Furthermore, we evaluated adverse events, including intraocular pressure instability, hemorrhage, retinal tears or detachment, and postoperative inflammation after these examinations. Cases 2, 3, 5, 6, 9, 19, and 20 were considered secondary VRL because the primary lesion was located outside the eye (Supplemental Table 1). Of the 22 cases included in this study, 15 cases were classified as primary VRL and seven cases as secondary VRL.

**Table 1.** Cytological findings of liquid-based cytology and cell block in this study

Case	Side	Primary lesion	Liquid-based cytology		Cell block obtained by vitrectomy under infusion								
			Atypical large lymphocytes	Necrosis	Atypical large lymphocytes	CD20	CD79a	CD10	BCL6	MUM1	CD3	CD5	Ki-67 LI (%)
1	Left	Eye	+	+	+	+	+	–	–	+	–	–	70
1	Right	Eye	+	+	+	ND	ND	ND	ND	ND	ND	ND	ND
2	Left	Testis	+	–	–	+*	ND	ND	ND	ND	ND	ND	ND
3	Left	Eye, CNS	+	+	+	+	+	ND	ND	+	ND	ND	70
4	Left	Eye	+	ND	ND	+*	ND	ND	ND	ND	ND	ND	ND
4	Right	Eye	+	ND	ND	+*	ND	–*	+*	+*	–*	–*	ND
5	Right	Lymph node	+	+	ND	+*	ND	ND	ND	ND	ND	ND	ND
6	Right	Lymph node	+	–	+	+	+	–	–	+	ND	ND	90
6	Left	Lymph node	+	+	+	+	+	–	+	+	–	–	70
7	Left	Eye	+	+	+	+	+	–	ND	ND	–	–	80
8	Left	Eye	+	+	+	+	+	–	ND	ND	–	–	80
9	Left	CNS	+	+	+	+	+	ND	+	ND	–	–	70
9	Right	CNS	+	–	–	ND	ND	ND	ND	ND	ND	ND	ND
10	Right	Eye	+	+	ND	ND	ND	ND	ND	ND	ND	ND	ND
10	Left	Eye	+	–	ND	ND	ND	ND	ND	ND	ND	ND	ND
11	Left	Eye	+	ND	ND	ND	ND	ND	ND	ND	ND	ND	ND
12	Right	Eye	+	+	+	+	+	–	–	+	–	–	70
13	Left	Eye	+	+	+	+	+	–	+	+	–	–	50
14	Left	Eye	+	+	+	+	ND	–	+	+	ND	ND	70
14	Right	Eye	–	+	+	+	ND	ND	+	-	ND	ND	60
15	Left	Eye	+	+	+	+	+	–	–	-	–	–	30
16	Right	Eye	+	–	+	+	+	ND	ND	ND	–	–	50
17	Left	Eye	+	+	+	+	+	–	+	+	ND	ND	70
18	Left	Eye	+	+	+	+	+	–	–	+	ND	ND	70
19	Right	Testis	+	+	+	+	+	ND	ND	ND	–	ND	70
20	Left	Liver, spleen	–	+	+	+	+	–	+	+	ND	ND	60
20	Right	Liver, spleen	+	+	+	+	+	–	–	+	ND	ND	60
21	Right	Eye	+	+	+	+	+	ND	ND	ND	ND	ND	70
22	Left	Eye	–	–	–	ND	ND	ND	ND	ND	ND	ND	ND

*, Since cell blocks could not be prepared or atypical lymphocytes were not observed in the cell block, immunocytochemical staining was performed using liquid-based cytology.

*CNS* central nervous system, *ND* no data

### Vitrectomy

Vitrectomy was performed to remove vitreous opacity associated with vision disorders in 29 eyes with VRL. All cases were treated using a 25-gauge pars plana vitrectomy with the EVA vitrectomy system (DORC) for making a definitive diagnosis of VRL, removal of vitreous opacity, as previously described [12]. Standard core vitrectomy was performed under infusion with BSS Plus™ (Alcon), and diluted vitreous fluid filling the tube of the vitrectomy cutter was obtained for LBC while observing the surgical field, target areas rich in cellular components. Using the same method, samples for flow cytometry and gene rearrangement were also collected in order. After the vitrectomy, diluted vitreous cell components in the drainage bag (200-300 ml) were collected for cell block.

This study was approved by the Research Ethics Committee of Yamagata University Faculty of Medicine (2019-314) and performed in accordance with the Declaration of Helsinki. Because this study was retrospective, the requirement for obtaining informed consent from the patients was waived by the institutional review board.

### LBC and cell block

**Fig. 2:**
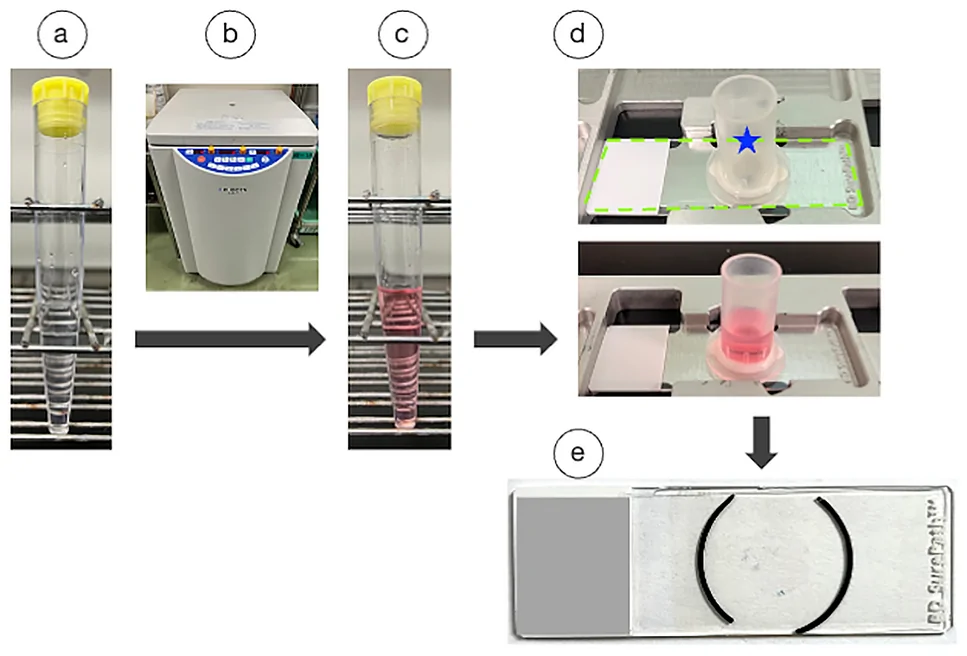
The preparation procedure of liquid-based cytology (LBC) (a and b) A total volume of about 2.0 ml of the vitreous specimen in the vial **(a)** was centrifuged (Kubota) at 3000 rpm for 5 minutes **(b)**, and the supernatant was discarded. **c.** The sediment was diluted with Cytorich Red Preservative (BD Diagnostics) at a ratio of 1:3. A total volume of about 2.0 ml of red sample. **d.** We attached the precoat slide (*A light green dashed trapezoid*) and the settling chamber (*A blue star*) to the BD Total Slide Tray (BD Diagnostics). We poured red sample to the settling chamber and allowed to incubate at room temperature for 15 min. **e.** The precoat slide was fixed in 95% ethanol at 10 min for Papanicolaou staining. Solutions of a, c, and d were samples.

LBC was performed by targeting areas with a high cell concentration while observing the surgical field under infusion. We performed LBC on 29 samples from vitreous bodies to identify atypical large lymphoid cells in patients with VRL (Table 1), as previously described [12, 13]. A total volume of 2.0 ml of vitreous in the vial was obtained just after creation of the three ports required for the procedure, which was completed without infusion. The vitreous body samples were treated at room temperature. LBC was performed using a SurePath^TM^ (BD Diagnostics) system (Fig. 2). We centrifuged (KUBOTA) it at 3,000 rpm for 5 minutes, then discarded the supernatant. The sediment was diluted with CytorichTM Red Preservative (BD Diagnostics) at a ratio of 1:3. We attached the precoat slide and the settling chamber to the BD Total Slide Tray (BD Diagnostics). A total volume of 2.0 ml of red sample was poured to the settling chamber and allowed to incubate at room temperature for 15 min. The precoat slides were fixed in 95% ethanol at 10 min for Papanicolaou staining. Immunohistochemical staining was performed by Simple Stain MAX PO (Nichirei) in some cases.

Cell block was performed by the vitreous cell components in the intraocular irrigating fluid/the diluted vitreous samples. The process of making cell blocks has been described previously [12]. After the supernatant liquid was eliminated, the sediment was fixed in 10% neutral-buffered formalin, five times the volume of the sediment, for 24 hours. After centrifugation at 3,000 rpm for 5 min (Model 5911, Kubota), the formalin was eliminated. We added 0.1 ml of 1% sodium alginate (Junsei chemical) and 0.2 ml of calcium chloride (Fujifilm Wako Pure Chemical Corporation) to the sediment to make it solid. The solid sediment was embedded in paraffin and used for hematoxylin-eosin staining. Cell blocks were prepared for 29 specimens, and cytological findings were evaluable in 23 specimens (79.3%). In the remaining six specimens, cytological findings could not be evaluated because the number of cells contained was very low or absent (20.7%). Using these 23 cell blocks we diagnosed VRL by hematoxylin-eosin staining and/or immunohistochemistry (Table 1 and Supplemental Table 1). The antibodies were used for immunohistochemistry as follows; CD20 (L26, Agilent Technologies), CD79a (JCB117, Leica Biosystems), CD10 (56C6, Leica Biosystems), BCL6 (LN22, Leica Biosystems), MUM1 (EAU32, Leica Biosystems), CD3 (LN10, Leica Biosystems), CD5 (4C7, Leica Biosystems), and Ki-67 (MM1, Leica Biosystems).

Cytological and pathological diagnoses were made at Yamagata University Hospital between 2015 and 2025. All cases were reviewed by experienced pathologists (OR, Kabasawa T, and SK).

### IL-10/IL-6 ratio, flow cytometry, and gene rearrangement

To measure the quantity of cytokines, pure aqueous humor was collected in all cases before vitrectomy. The levels of IL-6 and IL-10 in the aqueous humor were measured by enzyme immunoassay. A ratio of IL-10/IL-6 > 1 was considered positive for supporting the diagnosis of VRL. Flow cytometry and gene rearrangement were also performed after cytology, targeting areas with a high cell concentration while observing the surgical field under infusion. Flow cytometry confirmed light chain restriction by the values of Igκ (polyclonal/FITC, Agilent Technologies) and Igλ (polyclonal/RPE, Agilent Technologies) of the diluted vitreous body. Polymerase chain reaction was conducted to confirm IgH, Igκ, and Igλ gene rearrangements.

## Results

### Safety of our biopsies of VRL

Our vitreous sample collection from 29 eyes of 22 patients, utilizing LBC and cell blocks with irrigation fluid, did not result in adverse events, including intraocular pressure instability, hemorrhage, retinal tears or detachment, and postoperative inflammation.

### LBC and cell block

**Fig. 3:**
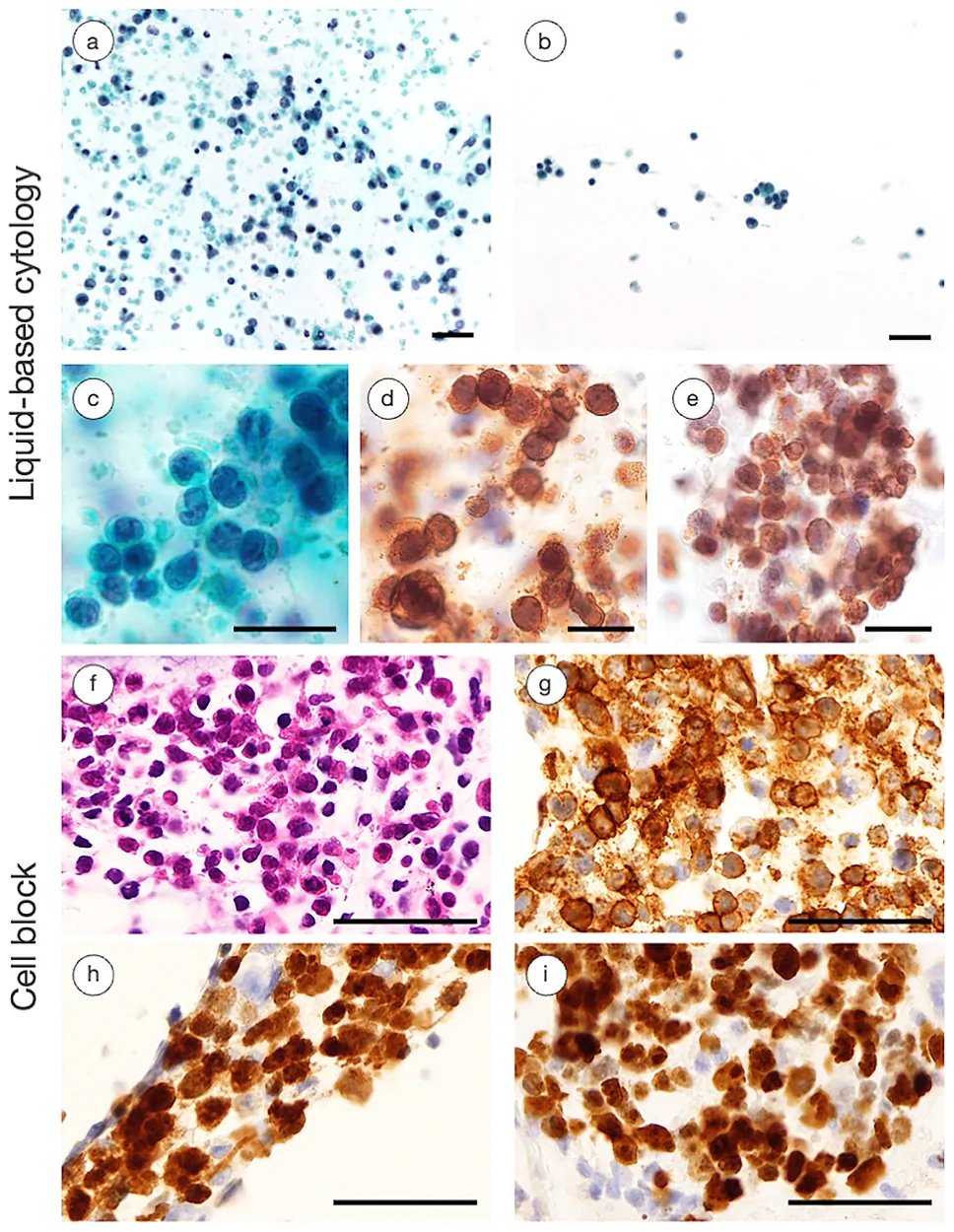
Cytopathological findings in this study (a-e) Liquid-based cytology (LBC) samples of the vitreous body obtained from a patient with vitreoretinal lymphoma (VRL). **a.** Necrosis is observed with numerous atypical large lymphocytes. Necrotic cells lose their nuclei and are stained light green. **b.** The appearance of atypical large lymphocytes without necrosis is also observed. **c.** Atypical large lymphoid cells, with a high N/C ratio, a notch in the nucleus, and two to three distinct nucleoli. **d.** Atypical large lymphoid cells are positive for CD20. **e.** The Ki-67 labeling index of this sample is 70%. **(f-i)** Cell block samples of the vitreous body obtained from a patient with VRL. **f.** Atypical large lymphoid cells, with a high N/C ratio and hyperchromatic nuclei, are clustered. **g.** Atypical large lymphoid cells are positive for CD20. **h.** Atypical large lymphoid cells are positive for MUM1. **i.** The Ki-67 labeling index of this sample is 90%. Bars, 50 μm.

LBC samples of the vitreous body were obtained from patients suspected of VRL. Necrosis was observed in 76.9% (20/26 samples) in the background of LBC samples (Fig. 3a, b). LBC samples showed atypical large lymphoid cells, with a high N/C ratio, a notch in the nucleus, and two to three distinct nucleoli (Fig. 3c). IHC of LBC samples showed that atypical large lymphoid cells are positive for CD20 (Fig. 3d) and high Ki-67 labeling index (LI) (Fig. 3e). In these LBC samples, atypical large lymphoid cells were identified in 89.7% (26/29 samples) (Table 1). These samples contained between 30 and over 10,000 atypical large lymphoid cells.

Cell block samples of the vitreous cell components were obtained from the intraocular irrigating fluid under infusion. Cell block samples showed atypical large lymphoid cells with a high N/C ratio and hyperchromatic nuclei that were clustered (Fig. 3f). IHC of cell block samples showed that atypical large lymphoid cells are positive for CD20 (Fig. 3g), MUM1 (Fig. 3h), and have a high Ki-67 LI (Fig. 3i). In these cell block samples, atypical large lymphoid cells were identified in 87.0% (20/23 samples) (Table 1). For each sample, clusters consisted of 10 or more lymphoid cells. Immunohistochemical staining revealed the following immunophenotypes for the lymphoid cells: CD20 (100%, 23/23 samples), CD79a (100%, 17/17 samples), CD10 (0%, 0/14 samples), BCL6 (57.1%, 8/14 samples), MUM1 (85.7%, 12/14 samples), and Ki-67 LI 66.3±12.7% (30-90%).

Taken together, we identified atypical lymphoid cells as lymphoma cells in 96.6% of samples (28/29) suspected of VRL based on results from LBC and cell block analysis. However, no pathological findings characteristic of VRL were observed in one sample, in which the lesion was subretinal and vitreous opacities were minimal (case 22). The history of steroid medication prior to sample collection did not affect the diagnosis for VRL (n=17; case 1-7, 9-15, 18, 20, and 22) (Table 1 and Supplemental Table 1).

In addition, no pathological findings characteristic of VRL were observed in 32 eyes suspected of ocular sarcoidosis, and in 45 eyes classified as other. Therefore, when our method was used to differentiate between 29 eyes suspected of VRL, 32 eyes suspected of ocular sarcoidosis, and 45 eyes classified as others, it achieved a sensitivity of 96.6% (28/29), a specificity of 100% (77/77), and a false positive rate of 0% (0/77).

### IL-10/IL-6 ratio, Flow cytometry, and gene rearrangement

The summary of the examination findings is shown in Supplemental Table 1. A ratio of IL-10/IL-6 > 1 was satisfied in 63.0% (17/27 samples). In flow cytometry, light chain restriction was observed in 53.6% (15/28 samples). IgH, Igκ, and Igλ gene rearrangements were detected in 89.7% (26/29 samples). In all cases, at least one of these three examinations showed findings suggestive of VRL.

## Discussion

The major method for obtaining pure vitreous samples involves performing a vitreous biopsy with scleral indentation [6] without irrigation. This method makes it challenging to control intraocular pressure and carries a risk of vision disorders in the affected eye. Thus, if a definitive diagnosis of VRL becomes possible using vitreous samples obtained during vitrectomy under infusion, which is a new procedure, different from the conventional method, the risk of complications to the affected eye would be decreased. In general, LBC is considered to provide high-quality cell fixation and to emphasize cell-nuclear details [7]. Furthermore, it is reported that LBC obtained from fine needle aspiration or endobronchial ultrasound-guided transbronchial needle aspiration provide higher sensitivity and specificity compared to conventional smears [8, 9]. In our institution, the qualitative diagnostic method utilizing LBC with irrigation fluid enabled the safe collection of vitreous samples [13]. This method proved useful for diagnosing VRL, vitreous amyloidosis, and sarcoid uveitis [14]. Furthermore, the addition of the cell block method enabled the identification of epithelioid cells/epithelioid granuloma in vitreous specimens from patients with sarcoid uveitis [12]. In this study, our vitreous sample collection from 29 eyes of 22 patients, utilizing LBC and cell blocks with irrigation fluid, did not result in adverse events. Furthermore, our method under infusion enabled the collection of lymphoma cells of sufficient quality and quantity for definitive diagnosis of VRL (Fig. 3 and Table 1). Therefore, our method of collecting vitreous samples, which maintains constant intraocular pressure under infusion was safe, yielded pure aqueous humor for cytokine measurement, and allowed biopsy of cells from parts with high cell density while visualizing the surgical field.

Morphological findings in VRL, which are large B-cell lymphomas, showed atypical lymphocytes in 100% (8/8) of LBC samples [15]. In cell block samples, atypical lymphocytes were observed in 91% (10/11) of cases, and necrosis was present in 100% [10]. In immunocytochemical findings of LBC and cell block samples in VRL, which were large B-cell lymphomas, demonstrated immunophenotypes; CD20 (91-100%), CD10 (0%), BCL6 (44-67%), and MUM1 (67-89%) [11] [15]. MUM1 is known to be a nuclear markers and recognizes the pre-plasmablast, plasmablast, and early plasma cells [16]. In our study, LBC was useful for identifying atypical large lymphoid cells and necrosis. Immunohistochemical staining was relatively easy to perform on cell block specimens prepared from the intraocular irrigating fluid/the diluted vitreous samples. CD20 and CD79a were important for confirming the B-cell phenotype. Furthermore, MUM1 was also a nuclear-staining marker and appeared suitable for use in over 85% of VRL cases. Additionally, over 60% of Ki-67 LI was found to be helpful in diagnosing VRL. CD10 was negative for VRL cases. Taken together, these findings suggest that our diagnostic method of VRL under infusion was equivalent to conventional methods and could serve as a definitive diagnostic tool for VRL.

Prior to diagnosing VRL, it was recommended to refrain from steroid medication because this medication was demonstrated to potentially exhibit cytotoxicity against lymphoma cells [17]. In this study, in 17 cases with steroid medication prior to vitreous biopsy, sufficient evidence to diagnose VRL was obtained in 94.1% cases (16/17). These results suggest that preoperative steroid medication does not necessarily affect VRL diagnosis.

We acknowledge a limitation of this study. One case showed primarily subretinal lesions with minimal vitreous opacity. In this case, atypical large lymphoid cells could not be identified via the LBC sample or cell block, rendering this diagnostic method ineffective. However, gene rearrangement was detected in IgH, Igκ, and Igλ in this case. Therefore, a negative LBC sample or cell block finding did not exclude VRL. Scarcity of lymphoma cells in the vitreous samples used for creating cell blocks indicates a limitation of our method. In this study, we measured the IL-10/IL-6 ratio using pure aqueous humor. To improve diagnostic accuracy, we plan to conduct a study that will measure the vitreous IL-10/IL-6 ratio using the supernatant obtained after centrifuging vitreous samples. In addition, *MYD88* L265P mutation, which contributes to the diagnosis of VRL, was not investigated.

In conclusion, the method employed in this study, which involves harvesting vitreous samples while visualizing the surgical field and preparing LBC and cell blocks with irrigation fluid, could help maintain stable intraocular pressure. This ensures safety and high diagnostic accuracy, thereby contributing to the definitive diagnosis of VRL.

## Supplementary Information

Below is the link to the electronic supplementary material.

Supplementary file1 (DOCX 24 kb)
