# Supplementary material for: Utility of liquid-based cytology and cell block obtained by vitrectomy under infusion to diagnose vitreoretinal lymphoma
Source: Jpn J Ophthalmol. 2026 Jun 30;70(5):1271–7. doi: 10.1007/s10384-026-01389-2 (PMC13615911; doi:10.1007/s10384-026-01389-2)
Supplement: Supplementary file 1 [file 10384_2026_1389_MOESM1_ESM.docx]

| Supplemental Table 1. Clinical findings of the patients in this study | | | | | | | | |
| --- | --- | --- | --- | --- | --- | --- | --- | --- |
| Case | Age | Sex | Side | IL-10/IL-6 | Flow cytometry  Light chain restriction | Gene rearrangement | Medication prior to vitrectomy* | Duration** (days) |
| 1 | 82 | Male | Left | 92.4 | - | IgH | BV | 21 |
| 1 | 82 | Male | Right | 136.1 | ND | IgH | Same as above |  |
| 2 | 82 | Male | Left | 10.8 | - | - | BV | 42 |
| 3 | 84 | Female | Left | 0.4 | κ | IgH | Flumetholon | 15 |
| 4 | 74 | Male | Left | 57.7 | κ | IgH, κ | BV | ND |
| 4 | 74 | Male | Right | ND | κ | IgH, κ | ND |  |
| 5 | 68 | Male | Right | 9.8 | - | IgH, κ | BV, Flumetholon | 316 |
| 6 | 70 | Female | Right | 0.5 | - | IgH, κ | BV, Flumetholon | 428 |
| 6 | 70 | Female | Left | 5.3 | κ | IgH, κ | Same as above |  |
| 7 | 73 | Female | Left | 8.8 | - | IgH, κ, λ | Prednisolone, BV | 1120 |
| 8 | 80 | Female | Left | 0.6 | λ | κ, λ | ND |  |
| 9 | 75 | Female | Left | 1.7 | κ | κ | BV | 31 |
| 9 | 79 | Female | Right | 1 | κ | κ | ND |  |
| 10 | 83 | Male | Right | 0.6 | - | κ | BV | 38 |
| 10 | 83 | Male | Left | 0.6 | - | κ | Same as above |  |
| 11 | 72 | Male | Left | 3.1 | κ | - | Flumetholon | 105 |
| 12 | 72 | Male | Right | 0.5 | λ | IgH, κ, λ | BV | 65 |
| 13 | 71 | Female | Left | 20 | κ | IgH, κ | Prednisolone, BV, STTA | 765 |
| 14 | 83 | Female | Left | 2.3 | - | IgH, κ | BV | ND |
| 14 | 83 | Female | Right | 1.7 | - | IgH, κ | Same as above |  |
| 15 | 58 | Male | Left | 1 | κ | IgH | BV | 11 |
| 16 | 82 | Male | Right | 0.1 | κ | - | ND |  |
| 17 | 59 | Male | Left | 5.3 | - | IgH | ND |  |
| 18 | 85 | Male | Left | ND | - | IgH, κ, λ | BV | 100 |
| 19 | 71 | Male | Right | 69.2 | κ | κ | ND |  |
| 20 | 74 | Male | Left | 5.5 | λ | κ, λ | BV | 46 |
| 20 | 74 | Male | Right | 3.2 | λ | κ, λ | Same as above |  |
| 21 | 84 | Female | Right | 1.6 | - | IgH, κ | ND |  |
| 22 | 68 | Male | Left | 0.7 | - | IgH, κ, λ | Flumetholon, STTA | ND |
| Igκ, κ; Igλ, λ; Betamethasone Valerate, BV; STTA, Subtenon triamcinolone acetonide injection; ND, no data; *, BV and Flumetholon were used as eye drops; **, Duration prior to vitrectomy | | | | | | | | |
